# Mechanical, Microstructure and Surface Characterizations of Carbon Fibers Prepared from Cellulose after Liquefying and Curing

**DOI:** 10.3390/ma7010075

**Published:** 2013-12-20

**Authors:** Xiaojun Ma, Cheng Yuan, Xinyan Liu

**Affiliations:** College of Packaging & Printing Engineering, Tianjin University of Science & Technology, Tianjin 300222, China; E-Mails: yzchen@tust.edu.cn (C.Y.); liuxinyan@tust.edu.cn (X.L.)

**Keywords:** cellulose, carbon fibers, surface characterization, mechanical properties, microstructure

## Abstract

In this study, Cellulose-based carbon fibers (CBCFs) were prepared from cellulose after phenol liquefaction and curing. The characteristics and properties of CBCFs were examined by scanning electron microscopy (SEM), Fourier transform infrared spectroscopy (FTIR), X-ray diffraction (XRD), Raman spectroscopy and X-ray photoelectron spectroscopy (XPS). The results showed that, with increasing carbonization temperature, the *L*_a_, *L*_c_, and *L*_c_/*d*_(002)_ of CBCFs increased gradually, whereas the degree of disorder *R* decreased. The –OH, –CH_2_–, –O–C– and phenyl group characteristic absorption peaks of CBCFs reduced gradually. The cross-linked structure of CBCFs was converted into a graphite structure with a six-ring carbon network during carbonization. The surface of CBCFs were mainly comprised of C–C, C–O, and C=O. The tensile strength, carbonization yield and carbon content of CBCFs obtained at 1000 °C were 1015 MPa, 52%, and 95.04%, respectively.

## 1. Introduction

Carbon fibers (CFs), a type of lightweight and high performance fibrous carbon material, are generally processed as reinforcement materials utilized in area where lightweight and high strength are required [1]. Because of their superior mechanical properties, they have potential application for reducing automobile weight and are receiving increasing attention as an important advancement in solving the urgent problem of reducing carbon dioxide. Cellulose-based carbon fibers were developed first in the 1950s and 1960s, and cellulose is the third largest source of carbon fibers after polyacrylonitrile (PAN) and pitch. Due to their low strength and low yield, cellulose-based fibers have been abandoned for high-strength applications in favor of PAN and pitch-based carbon fibers [2–6]. However, they are still widely used in ablative technology, as carbon fibers and other carbon textile products.

To meet the challenges posed by the rapid development of aerospace, transportation, and medical industries, serious research on cellulose-based fibers has focused on improving their mechanical properties and yield, and eliminating pollution problems [7–9]. Recently, biomass materials are almost completely converted into useful liquid chemical raw by liquefaction technique, which greatly improves the application of biomass materials [10]. It is reasonable to propose that liquefied cellulose is an excellent candidate for making high performance carbon fibers.

In this paper, cellulose-based carbon fibers were prepared successfully by liquefied cellulose, melt spinning, curing treatment, and carbonization. In order to a comprehensive understanding of cellulose-based carbon fibers, the molecular structure change, crystal structure transition, and surface functional groups of cellulose-based carbon fibers during carbonization were also investigated in detailed. At the same time, the mechanical properties, modulus and yield of cellulose-based carbon fibers at various temperatures were also compared.

## 2. Experimental

### 2.1. Samples

The mixture of 20 g of cellulose prepared by nitrate ethanol method [11] and 120 g of phenol as well as 9.6 g of H_3_PO_4_, loading in a round bottom flask, was heated in an oil bath at 160 °C for 150 min. the compound including cellulose and phenol is obtained. Subsequently, hexamethylenetetramine as synthetic agent was added to liquefied cellulose by 5 wt% (on the weight of liquefied cellulose) and was held for 5 min after heating to 180 °C in 40 min to prepare spinning solution. The spinning solution was placed into a spinning machine [12], and the spun filaments were prepared by melt-spinning. The spun filaments were cured by soaking in an acid solution HCHO and HCl (1:1 by volume) as main components at 95 °C for 4 h, washed with distilled water and finally dried at 90 °C for 45 min. The carbon fiber precursors from liquefied cellulose were prepared. The carbonization was carried out in a tubular furnace and the samples were heated from room temperature to the final carbonization temperature (400–1000 °C) with a heating rate of 3 °C/min in a 100 mL/min stream of N_2_. The samples were held for 60 min under the carbonization temperature, and then naturally cooled to room temperature. Cellulose-based carbon fibers (CBCFs) from liquefied cellulose were prepared.

### 2.2. Measurements

The cross-section of CBCFs was observed with a scanning electron microscope (S-4800, Hitachi Co., Tokyo, Japan).

The chemical structures of treated carbon fibers were detected at ambient temperature by a Fourier transform infrared spectrometer (FTIR) of TENSOR37 (BRUKER Co., Karlsruhe, Germany). The fibers were pulverized (150–200 mesh) and mixed with KBr before being pressed into a disk. The concentration of the sample in KBr was 2.5%, and 0.2 g of KBr was used in the preparation of the reference and sample disks.

The crystal structures were measured by scanning the fiber samples in the range of 5°–60° (2θ) by a Powder X-ray Diffractometer (D/MAX2500, Rigaku Co., Tokyo, Japan). The apparent crystallite thickness (*L*_c_), the apparent layer-plane length parallel to the fiber axis (*L*_a_), and the average interlayer spacing *d* were calculated using the Bragg and Scherrer formula. The formulas can be expressed as:

$$d=\frac{\lambda }{2\text{sin}\theta }$$ (1)

$$L=\frac{K\lambda }{\beta \text{cos}\theta }$$ (2)

where θ is the Bragg angle of peaks (°), λ is the wavelength of X-ray used (0.154 nm), and β is half-height width of peak (rad). The form factor K is 0.89 for *L*_c_, and 1.84 for *L*_a_, respectively.

The Raman scattering measurements were performed in a Raman spectrometer (Invia, Renishaw Co., Derbyshire, UK) at room temperature under a nitrogen atmosphere, using a 473 nm line of an argon ion laser as the incident radiation. The Raman spectrometer was operated in the continuous scanning mode with laser beam powers of 4 mW and exposure times of 25 s.

XPS measurements of the samples at various temperatures were carried out on a Kratos Axis UltraDLD multi-technique X-ray photoelectron spectroscopy (K-alpha, ThermoFisher Co., East Grinstead, UK) with a monochromated Al Kα X-ray source (*hv* = 1486.6 eV).

Tensile strength was measured by electrical tensile strength apparatus (YG004N, Hongda Co., Shanghai, china) under a span distance of 10 mm and a crosshead speed of 2 mm·min^−1^. The data shown are average value for 30 fibers.

## 3. Results and Discussion

### 3.1. Morphological Characteristics

**Figure 1.**
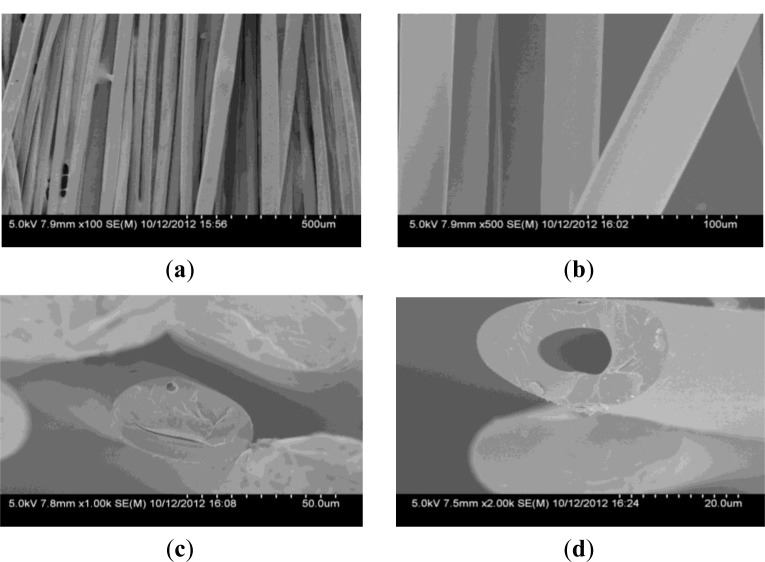
Scanning electron microscopy (SEM) micrographs of Cellulose-based carbon fibers (CBCFs): (**a**) and (**b**) side surface; (**c**) and (**d**) crossing section.

As can be seen from Figure 1a,b, the surface of CBCFs is smooth. The cross section shown in Figure 1c is elliptical pattern due to the squareness bobbin during melt- spinning process. Many fine pores are observed in the cross section, mainly around the center of the cross section (Figure 1d). It is due to the residual gas in spinning solution and the low crosslinkage degree of the inner of the carbon fiber precursors during curing treatment [13].

### 3.2. Mechanical Properties

**Figure 2.**
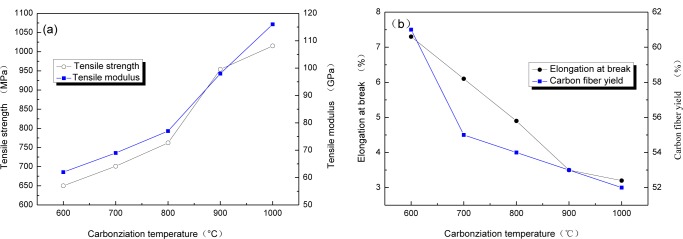
The mechanical properties of CBCFs: (**a**) Tensile strength; (**b**) Elongation at break and Carbon fiber yield.

The relationship between carbonization temperature and mechanical properties of CBCFs are reported in Figure 2. With carbonization temperature increasing, tensile strength and modulus of CBCFs obviously improve, while elongation at break rapidly reduces. Carbonization temperature increases from 600 to 1000 °C, tensile strength and modulus of CBCFs increase from 650 to 1015 MPa and from 62 to 116 GPa, respectively. At the same time, elongation at break decreases 54.9% from 600 to 1000 °C. From Figure 2a, it can also be seen that the mechanical properties of CBCFs are slightly better than those previously reported for carbon fibers produced from cellulose (tensile strength 0.8 GPa, modulus 70 GPa) [14].

Higher weight losses, or lower yield result in higher production costs [15]. Figure 2b shows the yields of carbon fibers are 61%~52% from 600 to 1100 °C. It is clear that the yield of CBCFs is higher than that of carbon fibers produced from cellulose.

### 3.3. Structure Transitions

**Figure 3.**
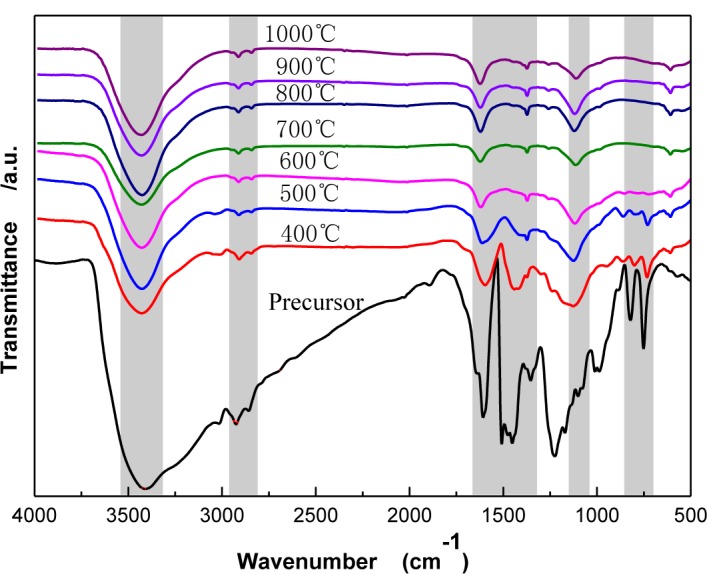
FTIR spectra of CBCFs at various temperatures.

Figure 3 shows Fourier Transform Infrared Spectroscopy (FTIR) spectra of CBCFs under different carbonization temperatures. As temperature increases, the wide-band absorption intensity of the associated hydroxyl group of CBCFs at 3427 to 3429 cm^−1^ is reduced, The absorption peaks at 2925 to 2850 cm^−1^ (mainly methylene bridge) and the absorption peak for the vibration of the benzene ring skeleton at 1631 to 1639 cm^−1^ weaken gradually. Moreover, the absorption peak of the stretching vibration at the C=C bond in the benzene ring skeleton at 1610 to 1504 cm^−1^ and the absorption peak of the C–O–C stretching vibration at 1124 to 1112 cm^−1^ are not changed after 500 °C. In addition, the two characteristic absorption peaks at 821 and 752 cm^−1^ almost disappear. This indicates that, due to the decrease of the –OH, –CH_2_, –O–C– and phenyl group during carbonization, the benzene rings of the sample get closer to each other, the benzene fused-ring structure is formed, and the cross-linked structure of the sample is changed into the six-member ring carbon network [16,17]. When carbonization temperature reaches 1000 °C, the absorption peak of the C=C bond (1610–1504 cm^−1^) of CBCFs do not disappear, indicating that the sample is difficult to graphitize [18].

### 3.4. Crystal Structure Transition

**Figure 4.**
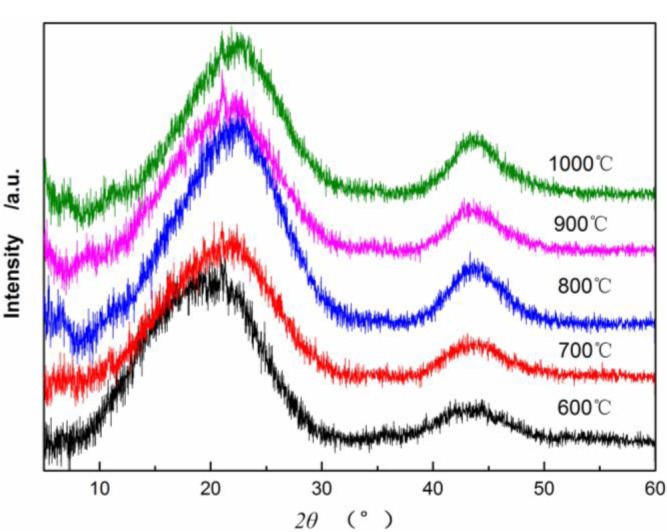
X-ray diffraction patterns of CBCFs at various temperatures.

Figure 4 shows X-ray diffraction patterns of CBCFs at various temperatures. As can be seen from Figure 4, the samples carbonized above 600 °C appear (002) and (100) peaks. As carbonization temperature increases, (002) peaks shift from 18.98° to 21.9°, which approaching the graphite (002) peak position, and (100) diffraction peaks enhance. This result indicates that carbon atoms of CBCFs are rearranged from disorderly to orderly and the crystalline structure improves remarkably with increasing carbonization temperature.

Table 1 shows that as carbonization temperature increases, the value of *d*_(002)_ decreases from 0.4670 to 0.4053 nm, whereas that of *d*_(100)_ decreases from 0.2073 to 0.2066 nm. The numerical variation of *d*_(002)_ is greater than that of *d*_(100)_, which is similar to the results of common carbon materials. However, the *d*_(002)_ of CBCFs is significantly greater than the *d*_(002)_ of graphite (0.3354 nm) and that of coke (0.3440 nm) at 1000–1350 °C [19,20]. This observation also indicates that, with increasing carbonization temperature, CBCFs is difficult to graphitize. In addition, the carbonization of CBCFs is quite complicated than the general carbon materials due to higher complexity of cellulose liquefaction. Meanwhile, *L*_c_, *L*_c_/*d*_(002)_, and *g* values (degree of graphitization) increase gradually as carbonization temperature increases, which indicates that the number of graphene sheets and the graphitization degree of CBCFs increase as temperature increases.

**Figure 5.**
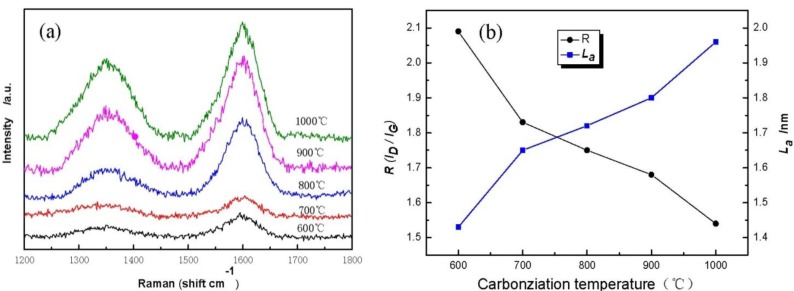
(**a**) Raman spectra of CBCFs at various temperatures; (**b**) the graphite crystalline size *L_a_* and *R* values of disorder.

Figure 5 displays Raman spectra of cellulose carbon fibers under different carbonization temperature. It is found that cellulose-based carbon fibers obtained above 600 °C exhibit D-peak at 1350 cm^−1^ (Figure 5a), which characterizes the disorderly structure of the carbon materials, and G-peak at 1597 cm^−1^, which characterizes the graphite crystallite structure of the carbon materials [21]. Peaks D and G are strengthened gradually as carbonization temperature increases, indicating thus that higher carbonization temperature could facilitate the arrangement of carbon from a disorderly to an orderly state [22,23]. Meanwhile, the existence of several oxygen-bearing functional groups affects the change in the mechanical constant of the C–C bond, resulting in the shift of curves D and G of the sample during the low-temperature carbonization stage. Figure 5b shows the graphite crystalline size *L_a_* and *R* values of disorder calculated using the Matthews’ formula and the Tuinstra-Koenig formula. with carbonization temperature increases, the *R* of the cellulose carbon fibers decreases, whereas *L_a_* increases, thus further indicating that the high carbonization temperature could improve the orderly state of the crystalline structure of the sample.

### 3.5. Surface Characterizations

**Figure 6.**
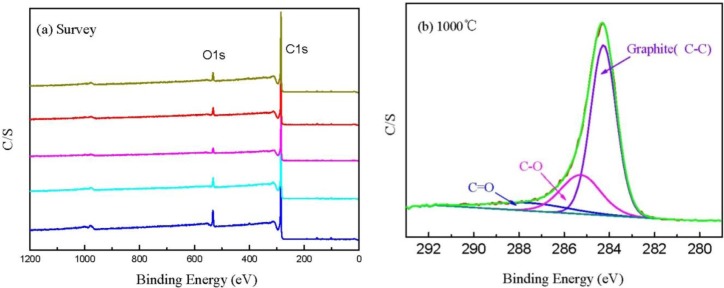
X-ray photoelectron spectroscopy (XPS) curve of CBCFs at various temperatures: (**a**) XPS survey spectra; (**b**) XPS spectra of the C1s region.

Figure 6a shows the XPS spectra of CBCFs at various temperatures. As shown in the figure, the elements C and O are the basic elements of CBCFs. The main elemental composition of the surface of CBCFs is shown in Table 2. As seen from Table 2, element C is the most abundant constituent of all CBCFs. With increasing carbonization temperature, elements C of CBCFs increases while elements O and N reduce. The elements C content of CBCFs have achieved more than 95% above 1000 °C.

In order to obtain information about the chemical composition of the fiber surface and the binding characteristics of the elements at the surface, measurements of the XPS spectra of the C1s region were analyzed. The C1s spectra of the five samples are almost the same, thus only the sample is shown in Figure 6b as an example. Figure 6b indicates that the C1s curve fitting is optimized into three independent peaks: the graphitic carbon (C–C, Binding Energy (BE) = 284.0–284.3 eV), either or hydroxy group (C–O, BE = 284.7–285.7 eV), and carbonyl or quinine groups (C=O, BE = 286.5–288.2 eV) [24,25]. The results of the fits of the C1s regions are listed in Table 3. It could be seen that the main peak of C1s corresponds to graphite carbon. With increased carbonization temperature, the contents of C–C bond increase and then decrease, while that of C–O and C=O bonds decrease, which is probably due to the removal of partly elements C and O during carbonization.

## 4. Conclusions

Although cellulose is the third largest source of carbon fibers, the poor strength and yield of cellulose-based carbon fibers has limited its applications. From the results reported in this study, cellulose-based carbon fibers with excellent performance and high yield were prepared after phenol liquefaction and curing. The high carbonization temperature could improve the orderly state of the crystalline structure of carbon fibers from liquefied cellulose, but carbon fibers from liquefied cellulose are difficult to graphitize. The results suggested that other wooden materials after liquefaction could become excellent raw materials for carbon fibers.

## Figures and Tables

**Table 1.** Structure parameters of X-ray diffraction for CBCFs.

Temperature (°C)	*d*_(002)_ (nm)	*d*_(100)_ (nm)	*L*_c_ (nm)	*L*_c_/*d*_(002)_	*g* (%)
600	0.4670	0.2073	0.6687	1.4318	−14.30
700	0.4237	0.2060	0.7090	1.6732	−9.27
800	0.4075	0.2062	0.7607	1.8665	−7.39
900	0.4193	0.2061	0.7265	1.7323	−8.76
1000	0.4053	0.2066	0.7937	1.9581	−7.13

**Table 2.** C, O, N contents of CBCFs at various temperatures.

Temperature (°C)	C (at%)	O (at%)	N (at%)
600	91.03	7.71	1.26
700	93.77	5.48	0.74
800	94.78	4.71	0.51
900	94.15	4.65	1.20
1000	95.04	4.58	0.38

**Table 3.** The surface functional groups content of CBCFs at various temperatures.

Temperature (°C)	Graphite(C–C)		C–O		C=O	

	BE (eV)	M (%)	BE (eV)	M (%)	BE (eV)	M (%)
600	284.0	56.62	284.7	29.59	287.5	13.79
700	284.2	73.47	285.7	8.59	286.5	17.94
800	284.2	72.07	285.4	25.19	288.2	2.75
900	284.3	68.46	285.3	23.21	287.3	8.23
1000	284.3	66.90	285.3	23.69	287.5	9.40
